# Medical Society of the County of Erie

**Published:** 1910-06

**Authors:** Franklin C. Gram


### SOCIETY PROCEEDINGS.

### Medical Society of the County of Erie

Reported by FRANKLIN C. GRAM, M. D., Secretary.

A REGULAR meeting of the Medical Society of the County of Erie was held, in the Buffalo Library Building, April 18, 1910, and was called to orded by the president, Dr. G. W. Wende, at 8.30 p. m.

On motion, the reading of the minutes of the previous meeting was dispensed with, for the reason that they had been published in the Buffalo Medical Journal.

Minutes of the council meetings of March 7, April 4, and April 16, were read by the secretary and, on motion of Dr. Lytle, were adopted and the recommendations contained therein approved.

Moved by Dr. Bonnar, seconded by Dr. Grant, that Dr. A. T. Bull be elected an honorary member of this society. Motion unanimously adopted.

Dr. T. H. McKee, chairman of the membership committee, then presented a list of thirty seven applicants for membership, each being individually balloted for and declared elected, as follows:

Ulysses B. Stein, 115 Franklin Street; George W. Grabenstatter, 155 Leroy Avenue; Bruce L. D. Cook, 819 Jefferson Street; George L. Fischer, 416 Sycamore Street; Samuel Kavinoky, 1061 Broadway; Robert K. Grove, 239 Delaware Avenue; Clarence L. Hyde, 1141 Genesee Street; Fred S. Hoffman, 547 Plymouth Avenue; A. W. Hengerer, 301 Genesee Street; Alfred F. Zittel, 611 Elm Street; Burton Thorne Simpson, 430 Delaware Avenue; Julien A. Riester, 230 E. Ferry Street; Alfred B. Wright, 603 Genesee Street; George C. Swerdfeger, 962 Jefferson Street; Frank C. Kleckner, 342 Monroe Street; Joseph A. Gregory, 2189 Seneca Street; James Hubert Donnelly, 16 West

Avenue; William E. Dignen, 490 Plymouth Avenue; Martin J. Downey, 852 Seneca Street; Harry N. Feltes, 136 Carlton Street; Edwin R. Gould, 160 Jefferson Street; Bennet T. Welch, 173 Prospect Avenue; Roland O. Meisenbach, 140 Allen Street; Irving R. Johnson, 403 Delaware Avenue; Max Keiser, 22 North Pearl Street; Charles A. Brownell, 204 Norwood Avenue; Edward E. Haley, 815 Niagara Street; F. Hubert Wilson, 739 Ellicott Street; W. H. Brauns, 355 Genesee Street; John B. Coakley, 339 Delaware Avenue; Julius A. Klahs, 256 E. Delavan Avenue; George W. Schaefer, 450 Genesee Street; Frank H. Ransom, Jr., 940 Jefferson Street; Charles J. Rosengren, 218 E. Ferry Street; Francis N. Pitass, 356 Peckham Street, Buffalo; Horace M. Edmonds, 46 Seymour Street, Tonawanda; Fred X. Fiegel, 103 Morgan Street, Tonawanda.

Dr. John H. Grant, Chairman of the Board of Censors, made report as follows:

Buffalo, April 18, 1910.

Memorandum report of the Board of Censors covering operations since report of February 21.

February 23. John Treskow convicted of practising medicine under an assumed name, that of the "Berlin Medical Offices," at 207 Main Street, and he was fined $50.00. The result of this was the closing of the place and its socalled anatomical museum. Treskow's case was reported to the Regents for the revocation of his license, and a committee of the State Board of Medical Examiners was convened in this city March 25, 1910, to hear and determine the case. Treskow's counsel appeared and said an appeal to the County Court had been made against the conviction and it would be heard during the May term. The Board of Examiners voted to revoke Treskow's license if the case was sustained by the County Court, otherwise the case stood dismissed.

March 7. The case against Mattie E. Lane for retrial was again postponed to the April term on account of the absence from the state of our principal witness. It will probably be tried, April 19.

March 8. One Ferdinand Schoenneman, of Hamburg, through the efforts of Dr. Fronczak, and the Board, was arrested for illegally practising medicine in East Buffalo. From one family he collected about $40.00 for prayers, herbs and salves at $3.00 per person. He was convicted and fined $200. This will be appealed on the ground that the criminal part of the City Court could not assess a greater fine than $50.00.

Another warrant in another case against Schoenneman has been sworn out, but he could not be found, though his counsel says he will appear after the $200 fine appeal has been decided. A new trial will probably be had in the former case and the fine reduced to $50.00.

The recovery, by the society, of the fines already imposed is held up by the appeals on the Treskow and Schoenneman cases. The $250 fine paid by Dellenbaugh will be paid to the Censors this week.

March 30. Your chairman investigated the R. A. Walker office at 79 Niagara Square. It was found that R. A. Walker was not registered in the County Clerk's Office; found a man posing there, in advertisements and to would-be patients, as Dr. R. A. Walker. To the chairman, of course, he denied he was Walker, but that he was a lawful practitioner, but refused to give the chairman his name upon request. I immediately swore out a warrant for his arrest for practising medicine under an assumed name. The case will be heard in City Court on May 4th.

Respectfully, for the Board of Censors,
John H. Grant, M. D., Chairman.

The foregoing report was received and approved.

Dr. J. D. Bonnar, Chairman of the Committee on Contagious Diseases Hospital, made the following report:

The members of the Committee on Hospital for Contagious Diseases, of the Medical Society of the County of Erie, conjointly with the committee for the same purpose of the Buffalo Academy of Medicine, having devoted two years to the work of providing such hospital for the city of Buffalo, are still unable to report completion of the purpose for which we were appointed. Nevertheless, while securing an appropriation therefor, strenuous opposition arising in vicinities where suitable sites were available, we are in statu quo, as regards location. While enjoying the unanimous belief of the medical profession, that the popular prejudice to the proximity of such hospital, is unwarranted, we are nevertheless unable to convince the public, thus far, that such a hospital is no menace to the health of the neighborhood, when constructed upon modern sanitary principles.

That the present temporary hospital on Broadway is not suited for such preventive service is indicated by the fact that the nurses must room out in the vicinity, and close beside their quarters several cases of scarlet fever exist, also one right opposite the hospital—suggestive, at least, of the danger of proximity to this ill-suited ramshackle structure, called a hospital.

While appreciating the faithful efforts of the councilmen in procuring a site, we deplore the failure, thus far, in reaching a decision thereon, by both boards of Council.

MINUTES OF COMMON COUNCIL.

School Examiners Report, 1905, Page 2728.

For years, great complaint has come from School 41, that the building could not be properly used by reason of the fact that the manufactory next door made so much noise that the teachers could not be heard in the rooms, nor could the pupils study. A lot for a new building was bought on Jefferson Street.

Arthur W. Hickman,
Isabella M. Holland,
John J. Hines,
E. Corning Townsend,
P. W. Van Peyma,
Board of School Examiners.

This state of affairs was corroborated at public hearings on site for hospital for contagious diseases, before the committee on sanitary measures of the Board of Aldermen, by Miss Bredell, a teacher, and by the principal of 41 School—old and new—and other teachers of the old 41. The building now being used for hospital was declared unsuited by the members of the two committees of the county society and academy of medicine, who jointly inspected it last summer, their report was then corroborated by its superintendent, Dr. Goodale. Mrs. Henry W. Altman, School Examiner, condemned it also.

Whereas, the Medical Society of the County of Erie, recognising the urgent necessity of a Municipal Hospital for Contagious Diseases deplores the unnecessary delay thus far displayed at the cost of the afflicted and the good name of the city by the authorities, and

Whereas, the present temporary hospital for the segregation and treatment of cases of acute communicable disease has been condemned by the Health Commissioner, the present Superintendent in charge, by a joint committee of the Medical Society of the County of Erie and the Buffalo Academy of Medicine and further that the site was condemned and abandoned for school purposes because of the noise from an adjoining factory in 1905 by the Board of School Examiners, and

Whereas, such a hospital, though of a temporary nature, cannot be conducted without danger to the community, be it

*Resolved,* That the Medical Society of the County of Erie again respectfully urges an immediate and definite action by the city authorities in the matter of acquiring a site, other than the Ferry street site, and the erection and maintenance of a modernly equipped hospital for the segregation and treatment of the acute communicable diseases.

*Resolved,* That such a hospital properly conducted is sanitary and by removing foci of infection a place of safety for the community, we recommend that a joint committee be appointed consisting of three members each, from the Board of Aldermen and the Board of Councilmen, also to be composed of the Board of Health (the Mayor, the Commissioner of Health and the Commissioner of Public Works) and that the Medical Society of the County of Erie and the Buffalo Academy of Medicine be represented by one member from each society to be appointed by the Mayor.

We recommend that this committee be given adequate power to solve this problem and to study the conditions in other cities where such hospitals already exist.

We recommend that copies of these resolutions be sent by the secretary to the Mayor, the President of the Board of Common Council, the President of the Board of Aldermen, the President of the Board of Councilmen, the Chairman of the Committee on Sanitary Measures, the Buffalo Academy of Medicine, the United Trades and Labor Council, the City Federation of Women's Clubs, the Chamber of Commerce and Manufacturers Club, and the Public Press."

The foregoing report of committee and resolutions contained therein were, on motion, adopted.

This concluded the business session and the following program was then carried out: The Importance of Urinary Examination in Children, by William Irving Thornton; Diphtheria Bacilli Carriers, by William G. Bissell; Inflammation of Verumontanum, with exhibition of instruments and cases, by James A. Gardner; Exhibition of Fillaria Sanguinis Hominis, by J. H. Potter and L. S. Beals; Routine Fecal Examination, by A. L. Benedict.

Discussions followed the presentation of the various papers.

The meeting then adjourned.

